# Silymarin Ameliorates Oxidative Stress and Enhances Antioxidant Defense System Capacity in Cadmium-Treated Mice 

**DOI:** 10.22074/cellj.2018.5355

**Published:** 2018-05-28

**Authors:** Elham Farjad, Hamid Reza Momeni

**Affiliations:** Department of Biology, Faculty of Science, Arak University, Arak, Iran

**Keywords:** Cadmium, Oxidative Stress, Silymarin

## Abstract

**Objective:**

Cadmium is an environmental pollutant which induces oxidative stress while silymarin as an antioxidant is able to scavenge free radicals. The aim of the present study was to investigate the effect of silymarin on oxidative stress markers and antioxidant defense system capacity in mice treated with cadmium chloride.

**Materials and Methods:**

In this experimental study, adult mice were divided into four groups as follow: i. Control, ii. Cadmium chloride (5 mg/kg b.w., s.c.), iii. Silymarin+cadmium chloride, and iv. Silymarin (100 mg/kg b.w., i.p.). Mice were treated with cadmium chloride for 24 hours and silymarin was administered 24 hours before the cadmium. Blood samples were then collected from the experimental groups and their sera were prepared. To investigate oxidative stress markers in the serum, the amount of malondialdehyde (MDA) and thiol groups (-SH) were evaluated. To measure the total antioxidant power in the serum, Ferric Reducing/ Antioxidant Power (FRAP) method was used. In addition, the activity of enzymes including catalase (CAT), superoxide dismutase (SOD) and glutathione peroxidase (GPx) was assessed to evaluate serum antioxidant defense power.

**Results:**

In the cadmium-treated group, the amount of MDA significantly increased as compared to the control group. In silymarin+cadmium group, silymarin significantly ameliorated the level of MDA compared to the cadmium group. In addition, cadmium significantly reduced serum FRAP, the activity of antioxidant defense system enzymes and thiol groups compared to the control. In silymarin+cadmium group, silymarin could significantly reverse the reduction of these markers compared to the cadmium group. Administration of silymarin alone caused a significant increase in serum FRAP, the activity of antioxidant defense system enzymes and thiol groups compared to the control group.

**Conclusion:**

Silymarin as a powerful antioxidant reverses the toxic effect of cadmium on the serum levels of lipid peroxidation, total antioxidant power, antioxidant defense system enzymes activity and thiol groups.

## Introduction

Cadmium is a heavy metal and an environmental pollutant; cadmium compounds are considered toxic and can accumulate in the body as well as the environment. Inhalation of cadmium vapors increases the level of this toxic element in the blood and thereby induces respiratory, liver and kidney cancers (1). Cadmium is used in batteries (particularly nickel-cadmium batteries), paint, coatings, electroplating, casting, refining, mining and as a stabilizer in plastics (2). Humans are also exposed to this pollutant through foods such as rice, wheat, fish, shell fish, drinking water and cigarette smoke (3).

In biological systems, generation of free radicals is inevitable and the body, with its antioxidant defense mechanisms, neutralizes, though not completely, the harmful effects of such free radicals. An imbalance between the free radicals production and the activity of antioxidant defense system enzymes leads to mild oxidative stress which is ameliorated by these enzymes (4). In severe conditions, however, oxidative stress damages cells, leading to cell death. Cadmium was documented to generate free radicals (5). This toxicant, due to its affinity to bind to sulfhydryl groups (thiol), deactivates antioxidants containing sulfhydryl groups (6). Furthermore, it is able to reduce antioxidant defense system enzymes including catalase (CAT), superoxide dismutase (SOD) and glutathione peroxidase (GPx) (5, 7).

Some medical plants are rich sources of antioxidants and consumption of such plants, by increasing the capacity of antioxidant defense system, could be a good strategy for eliminating the harmful effects of environmental pollutants, including cadmium. Silymarin is an effective substance extracted from the seed or the fruit of the medicinal plant *Silybum marianum* (8). This plant possesses several therapeutic effects such as anti- inflammatory, anti-anxiety, anti-hepatitis, anti-tumor, anti-cancer and neuroprotection (9). In addition, silymarin has potent antioxidant properties (10) and is able to remove free radicals and increase cellular glutathione content; also, as a membrane stabilizer, it can protect the cells against oxidative stress (8, 11).

Considering oxidative stress-inducing activity of cadmium, in this study, we investigated whether silymarin as a potent antioxidant can ameliorate the toxic effect of cadmium on oxidative stress markers and enhance antioxidant defense system capacity. 

## Materials and Methods

Silymarin was purchased from Sigma, USA. All other chemicals were purchased from Merck, Germany.

In this experimental study, adult male NMRI mice (30-35 g) obtained from Pasteur Institute, Tehran, Iran were used. The animals were housed in cages with 12 hours/12 hours light/dark cycle and had free access to water and food ad libitum. The experiments were approved by the local Ethical Committee at Arak University, Arak, Iran. The animals (n=24) were randomly divided into four groups as follow: i. Control, ii. Cadmium chloride (5 mg/kg body weight (12), as a single subcutaneous injection for 24 hours), iii. Silymarin+cadmium chloride [silymarin was injected 24 hours before the injection of cadmium chloride (13)], and iv. Silymarin (100 mg/kg body weight (14); as a single intraperitoneal injection for 24 hours). Cadmium chloride and silymarin were dissolved in saline and dimethyl sulfoxide (DMSO), respectively. Based on the solvents, two control groups namely, saline and DMSO, were selected. Since no significant difference was found between the results of the controls, saline group was considered as the control group. At the end of the treatments, the animals were anesthetized by injection of sodium pentobarbital (60 mg/kg) and blood samples were immediately obtained from the heart. Prepared sera were then kept at -80°C until used. The sera were used for the measurement of the levels of MDA, thiol groups, and total antioxidant power as well as antioxidant defense enzymes activity.

### Lipid peroxidation assay

Lipid peroxidation was evaluated by measuring the concentration of MDA. The reaction of aldehydes with thiobarbituric acid (TBA) produces a pink complex under acidic conditions at 100°C (15). Briefly, 600 µl of TBA solution [containing 15% (w/v) TBA, 0.375% (w/v) trichloroacetic acid (TCA) and 0.25 N hydrochlorid acid (HCl)] was added to 300 µl of the serum. The samples were incubated in a water bath at 95°C for 15 minutes and then chilled in ice. Finally, the samples were centrifuged at 1000 g for 10 minutes. The absorbance of supernatant was measured by a spectrophotometer (PG instruments T80 UV/VIS, UK) at 535 nm. The amount of MDA was calculated using its extinction coefficient (1.56×10^5^ M^-1^ cm^-1^) and expressed as nmol/ml (16).

### Assessment of serum total thiols 

The amount of thiol groups in the serum was assessed using the reduction of 2-2/-dinitro-5,5/-dithiodibenzoic acid (DTNB) reagent to create a yellow complex (17). Briefly, 250 µl Tris buffer (containing 0.25 M Tris base and 20 mM ethylene diamine tetra acetic acid (EDTA); pH=8.2), 25 µl of 10 mM DTNB and 1000 µl of absolute methanol were added to 25 µl of the serum. After 15 minutes of incubation at room temperature, the samples were centrifuged at 4000 g for 20 minutes and the absorbance of supernatant was measured by a spectrophotometer (PG instruments T80 UV/VIS, UK) at 412 nm. The amount of thiol groups was computed using extinction coefficient of the DTNB (13.6 mM) and expressed as mM (18).

### Measurement of total antioxidant power (FRAP method)

This method is based on the ability of serum in reducing ferric (Fe^3+^) to ferrous (Fe^2+^) by the action of electron donating antioxidants. Obtained Fe2+ produces a blue complex at acidic pH and in the presence of 2, 4, 6-tripyridyl-s-triazine (TPTZ) reagent (19). Briefly, 50 µl of serum was diluted with 50 µl of distilled water and then 900 µl of the FRAP reagent [containing 300 mM acetate buffer, pH=3.6, with 10 mM TPTZ in 40 mM HCl and 20 mM ferric chloride (FeCl_3_)] was added to the diluted serum. The reaction mixture was incubated in a water bath at 37°C and the absorbance was measured by a spectrophotometer (PG instruments T80 UV/VIS, UK) at 593 nm after 4 minutes. Different concentrations of iron sulphate were used for drawing a standard curve. The amount of FRAP was computed using a regression equation obtained from the standard curve and expressed as mmol/l (20). 

### Evaluation of the activity of antioxidant defense system enzymes

The activity of CAT was assessed according to Aebi method (21) which is based on the decomposition of hydrogen peroxide (H_2_O_2_) by CAT. Briefly, 2 ml of 50 mM potassium phosphate (K_3_PO_4_) buffer, pH=7, and 1 ml of 50 mM H_2_O_2_ were added to 50 ml of the serum and absorbance was ultimately measured by a spectrophotometer (PG instruments T80 UV/VIS, UK) at 240 nm between minutes 0 and 3. The activity of CAT was calculated based on an extinction coefficient for H_2_O_2_ (43.6 M^-1^ cm^-1^) and expressed as U/ml (12).

SOD activity was determined using a method described by Marklund (22). Pyrogallol was autoxidized rapidly in aqueous solution and employed for the estimation of SOD. Briefly, 2.8 ml of Tris buffer (containing 50 mM Tris buffer and 1 mM EDTA, pH=8.5) and 0.1 ml of 20 mM pyrogallol were added to 0.1 ml of the serum. The absorbance was read by a spectrophotometer (PG instruments T80 UV/VIS, UK) at 240 nm after 1.5 and 3.5 minutes as absorbance reading of control without serum=A and absorbance reading of sample with serum=B. The activity of SOD was measured using A-B/A×50 (100×10) formula and expressed as U/ml (23). 

The activity of GPx was assessed according to a method described by Rani and Unni KMKarthikeyan (24) with some modifications. This method is based on glutathione oxidation and reduction of H_2_O_2_ to water. In brief, 0.2 ml of 0.8 mM EDTA, 0.1 ml of 10 mM sodium azide (NaN_3_) and 0.1 ml of 2.5 mM H_2_O_2_ were added to 0.2 ml of the serum and incubated in a water bath at 37°C for 10 minutes. Then, 0.5 ml of 10% TCA was added to the reaction mixture and centrifuged at 2000 g for 15 minutes. Next, 3 ml of 0.8 mM disodium hydrogen phosphate (Na_2_HPO_4_) and 0.1 ml of 0.04% DTNB were added to the solution and the color intensity was measured by a spectrophotometer (PG instruments T80 UV/VIS, UK) at 420 nm. The activity of GPx was computed using extinction coefficient for DTNB (13600 mol/l) and expressed as U/L.

### Statistical analysis

Results were expressed as mean ± SD. One-way ANOVA followed by Tukey’s test was used to assess the statistically significant differences among the data using SPSS software, version 16 (IBM Co., USA). A P<0.05 was considered significant.

## Results

### Evaluation of lipid peroxidation 

**Fig.1:**
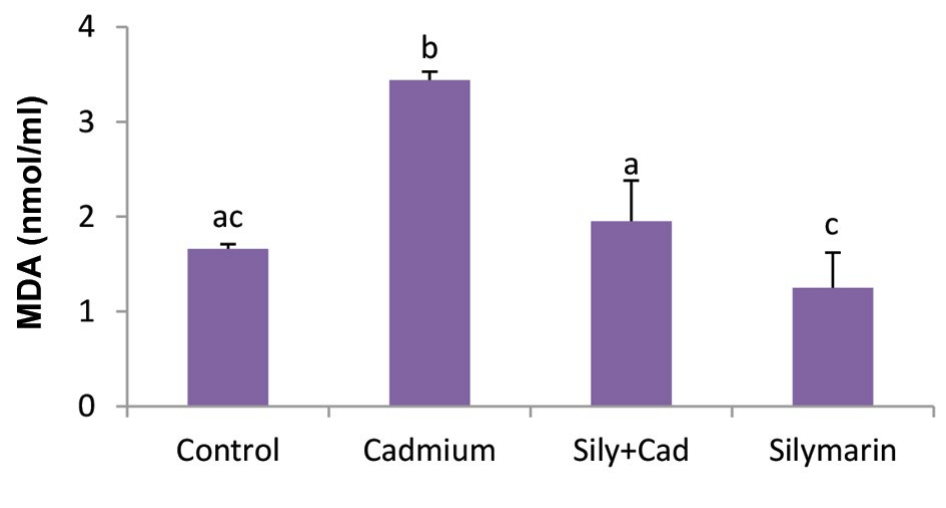
Evaluation of the level of serum malondialdehyde (MDA) in the groups treated with silymarin (100 mg/kg) and/or cadmium chloride (5 mg/kg). The data are expressed as mean ± SD. Different letters show significant differences as assessed by ANOVA followed by Tukey’s test (n=6, P<0.05).

In the cadmium chloride group, MDA level significantly (P<0.001) increased as compared to the control group. In the silymarin+cadmium chloride group, silymarin could significantly (P<0.001) reduce the level of MDA as compared to the cadmium chloride group (Fig .1).

### Evaluation of serum total thiols 

**Fig.2:**
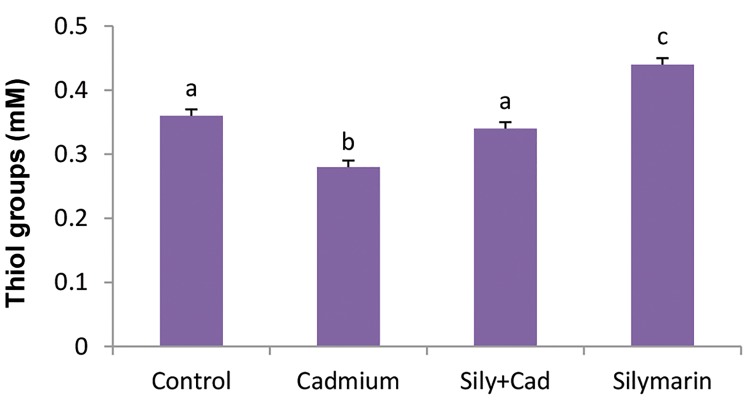
Evaluation of serum levels of thiol groups in the groups treated with silymarin (100 mg/kg) and/or cadmium chloride (5 mg/kg). The data are presented as mean ± SD. Different letters show significant differences as assessed by ANOVA followed by Tukey’s test (n=6, P<0.05).

In the cadmium chloride group, the level of the thiol groups showed a significant (P<0.001) reduction as compared to the control group. In the silymarin+cadmium chloride group, silymarin could significantly (P<0.001) ameliorate the level of thiol groups compared to the cadmium group. Also, treatment with silymarin alone for 24 hours caused a significant (P<0.001) increase in thiol groups level as compared to the control group (Fig .2).

### Evaluation of total antioxidant power (FRAP method)

**Fig.3:**
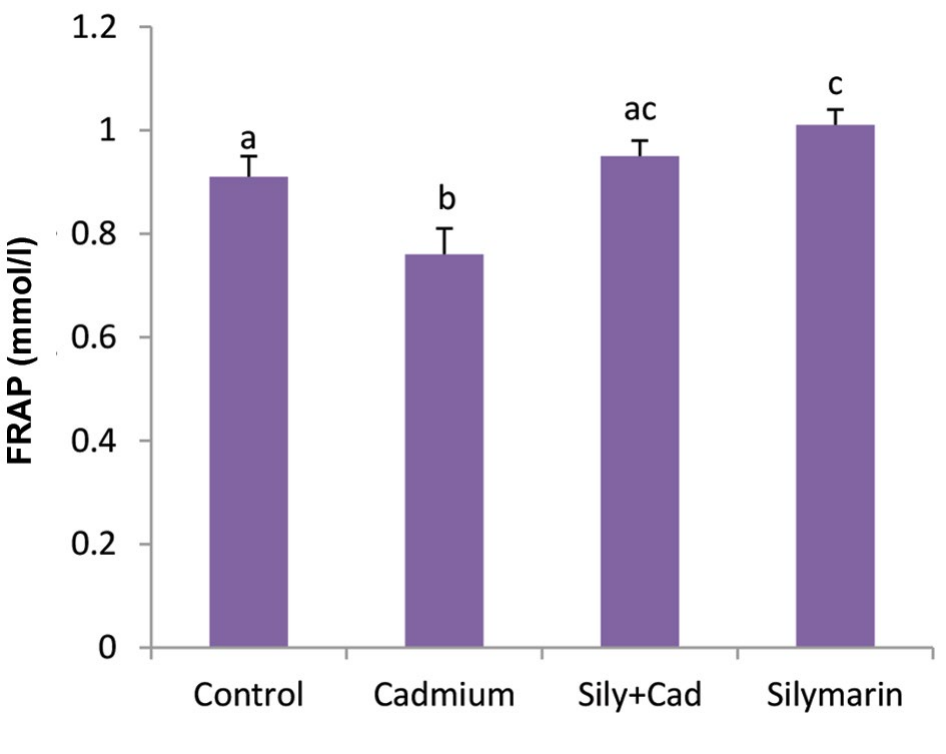
Evaluation of levels of serum Ferric Reducing/Antioxidant Power (FRAP) in the groups treated with silymarin (100 mg/kg) and/or cadmium chloride (5 mg/kg). The data are expressed as mean ± SD. Different letters show significant differences as assessed by ANOVA followed by Tukey’s test (n=6, P<0.05).

In the cadmium chloride group, the serum levels of FRAP were significantly (P<0.001) reduced as compared to the control group. In the siymarin+cadmium chloride group, silymarin could significantly (P<0.001) compensate the amount of the FRAP levels compared to the cadmium group. Treatment with silymarin alone for 24 hours significantly (P<0.001) increased FRAP levels compared to the control group (Fig .3).

### Evaluation of the activity of serum antioxidant defense system enzymes 

**Fig.4:**
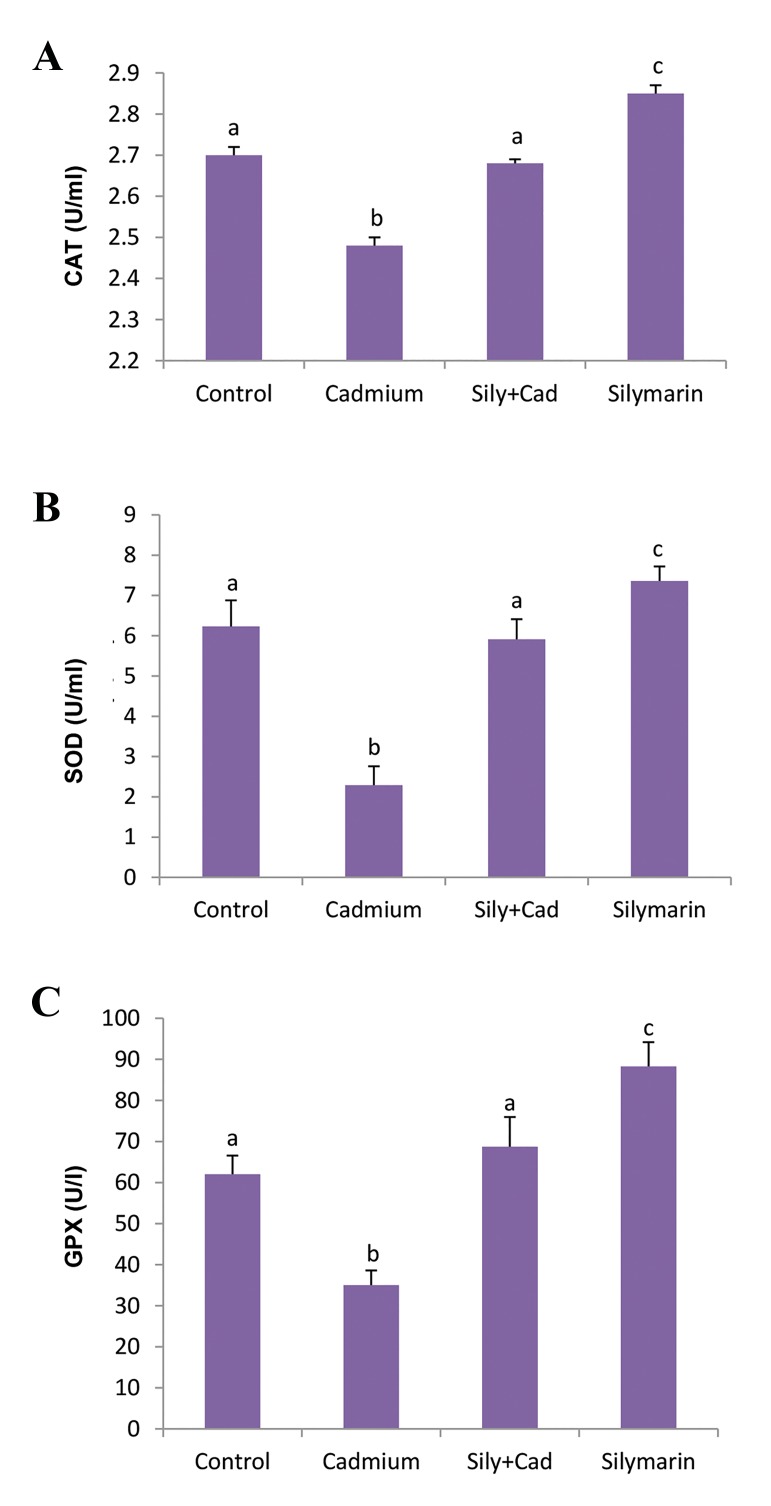
Activity of antioxidant defense system enzymes in the groups treated with silymarin (100 mg/kg) and/or cadmium chloride (5 mg/kg). A. Catalase (CAT), B. Superoxide dismutase (SOD), and C. Glutathione peroxidase (GPx). The data are presented as mean ± SD. Different letters show significant differences as assessed by ANOVA followed by Tukey’s test (n=6, P<0.05).

In the cadmium chloride group, the activity of serum CAT (Fig .4A), SOD (Fig .4B) and GPx (Fig .4C) was significantly (P<0.001) reduced as compared to the control group. In the silymarin+cadmium chloride group, silymarin could significantly (P<0.001) ameliorate the activity of these enzymes compared to the cadmium group. Also, administration of silymarin alone for 24 hours caused a significant (P<0.001) increase in the activity of the enzymes as compared to the control group.

## Discussion

This study showed that cadmium chloride as an environmental pollutant exerts detrimental effects on lipid peroxidation, serum total thiols and serum antioxidant defense system while silymarin, as an antioxidant could reverse the damaging effects of cadmium chloride on these markers. 

One of the adverse effects of oxidative stress is induction of lipid peroxidation (25) and reduction of serum total thiols (26) which have damaging effects on cells and tissues. In the present study, we showed that cadmium chloride increased MDA and decreased thiol groups in the serum. In addition, this environmental pollutant not only reduced the activity of serum antioxidant defense system enzymes including CAT, SOD and GPx but also reduced serum total antioxidant power (measured by FRAP).

Cadmium can exert its destructive activity through induction of oxidative stress through at least two ways, the first of which is the generation of free radicals. One of the mechanisms involved in this case is that cadmium can replace with Fe in various membrane and cytoplasmic proteins such as ferritin and apoferritin, thus, increases the amount of freely available Fe ions that participate in Fenton reaction and generate free radicals (27). In addition, cadmium binds to cysteine in glutathione to reduce thiol groups and alters its activity resulting in production of free radicals (16). The radicals react with polyunsaturated fatty acids (PUFAs) leading to lipid peroxidation. MDA is the end product of lipid peroxidation and an indicator of the induction of oxidative stress (28). 

Cadmium increases the production of superoxide anion radicals and thereby can convert ferric (Fe3+) to ferrous (Fe2+) to produce hydroxyl radicals via the Fenton reaction, which in turn increases serum oxidative stress levels (29). The second way, through which cadmium can play its destructive role in the induction of oxidative stress, is through reducing the activity of antioxidant defense system enzymes. Cadmium, through interaction with the elements such as zinc, copper and manganese in the SOD molecule, inhibits the activity of this enzyme (30). Decreased SOD activity may reduce H_2_O_2_production followed by a decrease in the activity of CAT, an enzyme which catalyses the conversion of H_2_O_2_ to H2O and molecular oxygen (31). Moreover, cadmium, through reaction with selenium in the GPx molecule, inactivates this enzyme and thus, reduces the decomposition of H_2_O_2_to the water (29).

Based on the central role of cadmium in the induction of oxidative stress (16, 32), it is likely to assume that cadmium, by inducing oxidative stress, increased lipid peroxidation and caused a reduction in serum levels of total thiols and antioxidant defense system activity. If this hypothesis is true, the use of an antioxidant should ameliorate the toxic effects of this pollutant on these factors. In the present study, we found that in silymarin+cadmium chloride group, silymarin could reverse the adverse effects of cadmium chloride on lipid peroxidation, serum total thiols, antioxidant defense system enzymes activity and total antioxidant power in the serum. Silymarin as a potent antioxidant (8) is able to scavenge free radicals (11) and increase the capacity of antioxidant defense system (9) in the cells and tissues. It is a polyphenolic compound and the presence of a methoxy group on its phenolic ring increases its antioxidant properties (33). Furthermore, silymarin, through increasing the level of phosphorylation at specific serine and/or tyrosine residues of nuclear factor erythroid 2-related factor 2 (NRF2), induces the expression of antioxidant proteins namely, antioxidant defense system enzymes and thiol molecules (34).

The findings of this study also showed that silymarin alone increased total thiols, antioxidant defense system enzymes activity and total antioxidant power in the serum compared to the control group. These results could support our hypothesis that silymarin with its antioxidant properties and through boosting the antioxidant defense system, reduces oxidative stress.

## Conclusion

Cadmium is an environmental pollutant which increases lipid peroxidation and reduces serum total thiols as well as the capacity of serum antioxidant defense system by inducing oxidative stress. However, silymarin could reverse harmful effects of this pollutant in terms of oxidative stress markers.
